# The association between humidex and tuberculosis: a two-stage modelling nationwide study in China

**DOI:** 10.1186/s12889-024-18772-8

**Published:** 2024-05-11

**Authors:** Wen Li, Jia Wang, Wenzhong Huang, Yu Yan, Yanming Liu, Qi Zhao, Mingting Chen, Liping Yang, Yuming Guo, Wei Ma

**Affiliations:** 1 Department of Epidemiology, School of Public Health, Cheeloo College of Medicine, Shandong University, Jinan, Shandong China; 2 Shandong University Climate Change and Health Center, Jinan, Shandong China; 3 National Center for Tuberculosis Control and Prevention, Chinese Center for Disease Control and Prevention, Beijing, China; 4 Climate, Air Quality Research Unit, School of Public Health and Preventive Medicine, Monash University, Melbourne, Australia

**Keywords:** Tuberculosis, Humidex, Time-series study, Exposure-response relationship

## Abstract

**Background:**

Under a changing climate, the joint effects of temperature and relative humidity on tuberculosis (TB) are poorly understood. To address this research gap, we conducted a time-series study to explore the joint effects of temperature and relative humidity on TB incidence in China, considering potential modifiers.

**Methods:**

Weekly data on TB cases and meteorological factors in 22 cities across mainland China between 2011 and 2020 were collected. The proxy indicator for the combined exposure levels of temperature and relative humidity, Humidex, was calculated. First, a quasi-Poisson regression with the distributed lag non-linear model (DLNM) was constructed to examine the city-specific associations between humidex and TB incidence. Second, a multivariate meta-regression model was used to pool the city-specific effect estimates, and to explore the potential effect modifiers.

**Results:**

A total of 849,676 TB cases occurred in the 22 cities between 2011 and 2020. Overall, a conspicuous J-shaped relationship between humidex and TB incidence was discerned. Specifically, a decrease in humidex was positively correlated with an increased risk of TB incidence, with a maximum relative risk (RR) of 1.40 (95% CI: 1.11–1.76). The elevated RR of TB incidence associated with low humidex (5th humidex) appeared on week 3 and could persist until week 13, with a peak at approximately week 5 (RR: 1.03, 95% CI: 1.01–1.05). The effects of low humidex on TB incidence vary by Natural Growth Rate (NGR) levels.

**Conclusion:**

A J-shaped exposure-response association existed between humidex and TB incidence in China. Humidex may act as a better predictor to forecast TB incidence compared to temperature and relative humidity alone, especially in regions with higher NGRs.

**Supplementary Information:**

The online version contains supplementary material available at 10.1186/s12889-024-18772-8.

## Background

Tuberculosis (TB) is a contagious disease, caused by the bacillus *Mycobacterium tuberculosis*. TB is transmitted through the air when infected people cough or sneeze. TB ranked high among global deaths from a single infectious disease, surpassing HIV/AIDS [1]. In 2021, an estimated 10.6 million people were diagnosed with TB worldwide [2]. China has a substantial disease burden of TB and ranks third globally in the number of reported TB cases [1].

Climate change has emerged as a major threat to human health in the 21st century. Alterations in climate factors have a noticeable impact on the incidence and burden of TB [3, 4]. Numerous studies have assessed the relationship between climatic variables and TB incidence, with a focus on temperature and relative humidity. For instance, studies in Malaysia [5] and Brazil [6] have unveiled associations between TB prevalence and relative humidity and temperature. Several studies have also been conducted in specific cities and regions of China, but with conflicting results on whether temperature is positively or negatively correlated with TB incidence [7–9]. However, these studies have primarily focused on the impact of individual meteorological variables on the TB epidemic, without considering potential interactive effects between these variables.

Given that the human body is affected by a variety of meteorological conditions, and different levels of humidity can affect the human body’s heat tolerance threshold, studies on the effect of temperature or relative humidity alone on TB may not reflect the actual situation. Therefore, it is more essential and practical to develop a composite index to reflect the combined effect of temperature and humidity on TB incidence nationwide. Such an index would substantially contribute to the formulation of effective health control measures.

Humidex, an index devised by Canadian meteorologists J.M. Masterton and F.A. Richardson, is a numerical representation of the combined influence of temperature and relative humidity on human comfort and health [10]. It has been used in many health studies [11], such as those on childhood asthma [12] and bacillary dysentery [13]. Notably, it has been effectively applied in research within both urban and rural settings [11, 14]. For example, it was applied to assess the impact of heat exposure on traumatic injuries among outdoor agricultural workers in Washington State, encompassing both urban and rural areas [15]. However, to the best of our knowledge, there are currently no studies that specifically examine the impact of humidex on TB incidence.

This study aimed to evaluate the association between humidex and TB incidence using weekly data obtained from 22 Chinese cities during 2011–2020, assess the variability of this association across different genders, age groups, and regions, and evaluate the potential effect modifiers by using geographic factors, socioeconomic factors, demographic variables, and health service information.

## Methods

### Data collection

**Fig. 1:**
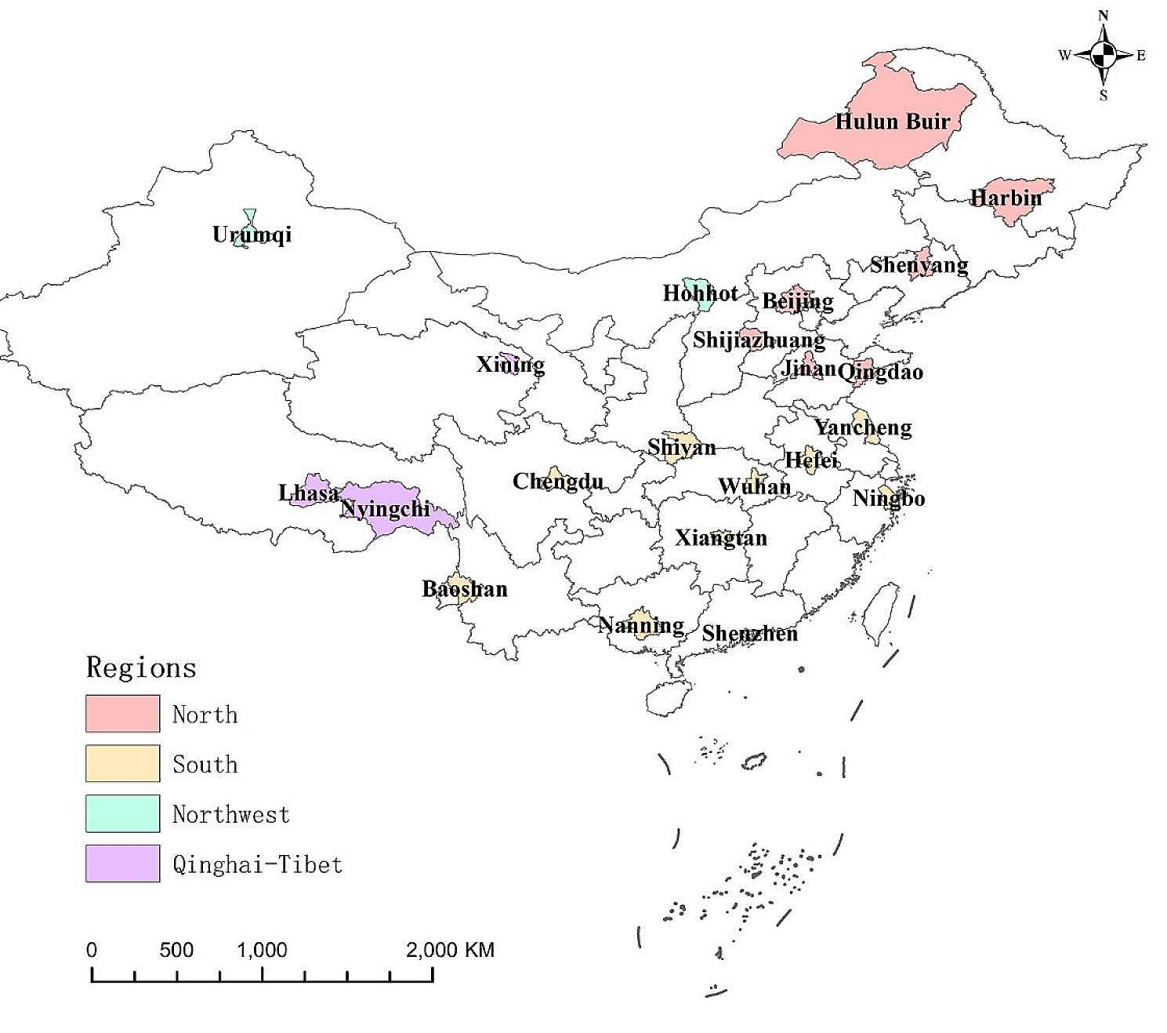
Geographic distribution and regions of 22 cities in China

This study included 22 cities from different meteorological geographical divisions in China and grouped these cities to four major geographical regions (North, South, Northwest, and Qinghai-Tibet area) for further exploration, according to geographical location, natural conditions, and social culture [16], including 7 cities in the North region (Beijing, Shijiazhuang, Hulun Buir, Shenyang, Harbin, Jinan, Qingdao); 10 cities in the South region (Yancheng, Ningbo, Hefei, Wuhan, Shiyan, Xiangtan, Shenzhen, Nanning, Chengdu, Baoshan); 2 cities in the Northwest region (Hohhot, Urumqi); and 3 cities in the Qinghai-Tibet region (Lhasa, Nyingchi, Xining) (Fig. 1, Table S1).

Weekly TB cases data for each city from 1 January 2011 to 31 December 2020, were collected from the Infectious Disease Reporting System and the Tuberculosis Information Management System, maintained by the Chinese Center for Disease Control and Prevention. Daily meteorological data for each city in the same period were obtained from the Atmospheric Reanalysis global climate dataset, maintained by the fifth-generation European Centre for Medium-Range Weather Forecasts [17], including daily mean temperature, relative humidity, mean wind speed. Daily humidex combined daily temperature and daily relative humidity into an index to indicate the perceived temperature (**Equation S1**). The daily mean concentration of fine particulate matter (PM_2.5_) was obtained from the Tracking Air Pollution in China [18]. Both daily meteorological data and PM_2.5_ data were transformed into weekly averages and then matched with TB case data.

Yearly data on various potential factors for each city from 2011 to 2020 were collected from the China City Statistical Yearbook [19], including geographic information (Latitude and Longitude), socioeconomic information (Gross Regional Product [GDP], and GDP Per Capita), demographic information (Population and Natural Growth Rate [NGR]), and health service information (Hospitals, Doctors, and Hospital Beds) (Table S1).

### Statistical analysis

To explore the association between humidex and TB incidence, a two-stage modelling strategy was adopted, which has been widely used in large-scale multi-city studies on environmental factors and health, including infectious diseases [20, 21]. In the first stage, we utilized a quasi-Poisson regression in conjunction with the distributed lag non-linear model (DLNM) to explore the Humidex-TB association for each city. This model not only accommodates non-linear exposure-response relationships but also considers lag effects [22], which allowed us to explore the city-specific associations between humidex and TB incidence.

The model could be expressed as follows:

1 $$\begin{array}{*{20}{c}}\begin{gathered}{\text{log}}\left[ {E\left( {{Y_{it}}} \right)} \right] = \hfill \\\,\,\,\,\,\,\,\,\,\,\,\,\,\,\,\,\,\,\,\,\,\,\,\,\,\,\,\,\,\alpha + cb\left( {humide{x_{it}}} \right) + ns\left( {w{s_{it}},df = 3} \right) + ns\left( {p{m_{it}},df = 3} \right) \hfill \\ \end{gathered} \\ {ns\left( {wee{k_{it}},df = 4} \right)} \end{array}$$

Where ${Y}_{it}$ is the observed weekly count of TB cases at the week *t* in the city *i*, α is the intercept; $\text{c}\text{b}$ is the cross-basis function; $humidex$ is the weekly mean humidex; nature cubic spline ($\text{n}\text{s}$) with 3 degrees of freedom ($df$) was used for weekly mean windspeed ($ws$) and weekly mean PM_2.5_ ($pm$) respectively; $ns\left({week}_{it},df=4\right)$refers to the nature cubic spline of the number of *week* at the week *t* in the city *i*, which is used to control the long-term trends and cyclical fluctuations [7, 23]. Taking into account findings from previous research indicating lag effects of meteorological factors on TB that can extend from 4 to 6 months [7, 24], we established the maximum lag time of 24 weeks for this study.

In the second stage, a multivariate meta-analysis was applied to pool the city-specific effect estimates derived from the first stage, including the non-linear overall association and the lag-response association. The multivariate meta-analysis fitted through a random-effect model by maximum likelihood was applied at both national and regional levels (North, South, Northwest, and Qinghai-Tibet region). The reference point for this analysis was the minimum TB risk humidex in each region. The heterogeneity was assessed by multivariate extension of Cochran’s Q test and the *I*^*2*^ statistic [25]. The value of *I*^*2*^ explained the difference between basic model and model included modifying factors. To explore the potential effect modifiers, the city-specific characteristics including geographic factors, socioeconomic factors, demographic variables, and health service information, were also introduced in the multivariate meta-regression with the likelihood ratio (LR) and Wald tests.

The effect of high and low combined exposure levels of temperature and humidity on TB incidence was defined as the relative risk (RR) with 95% Confidence Intervals (CI) at the 95th and the 5th humidex percentile. The lagged effect of 95th and the 5th humidex for each city were explored in the first stage and then were pooled at the national level. The stratified analyses were also applied by region (North, South, Northwest, and Qinghai-Tibet area), gender (male and female), and age group (≤ 14 years, 15–39 years, 40–64 years, and ≥ 65 years) through the same methods. To discern differences between groups, we utilized the multivariate Wald test. To justify the effect of humidex, we also constructed a model using mean temperature and mean relative humidity with the same composition and parameters.

To assess the robustness of the results, sensitivity analysis was performed by (a) altering the max lag time from 23 to 25 to test 24 weeks was suitable to capture the whole effect; (b) changing the *df* of time trend from 4 to 6 to determine whether 4 was sufficient to obtain the stable model; (c) the influencing factors (PM_2.5_, windspeed) were added to the model to test whether they influenced Humidex-TB relationships, respectively; (d) The number of cases in the previous week was added to test for its potential effect on the model.

All statistical analyses were performed in R software (version 3.6.2) with the ‘dlnm’ and ‘mvmeta’ packages. A two-sided *P* value < 0.05 was considered statistically significant.

## Results

From 2011 to 2020, there were 849,676 TB cases in the study area. Table 1 summarizes TB cases and meteorological variables in the 22 cities by region, gender, and age group. Males had more than twice the number of TB cases as females. The 15–40 age group had the most TB cases, accounting for 41.2% of the total, followed by the 40–65 age group (38.7%), the 0–15 age group (0.7%), and the > 65 age group (19.4%). The average mean weekly humidex at the national level was 12.66, ranging from − 37.72 to 46.83. Beijing and Shenzhen had higher GDP and GDP per capita, large cities such as Beijing and Chengdu had more hospital doctors and beds, while Nyingchi, Lhasa and Nanning had higher NGRs. See Table S1 for a description of city-specific characteristics.

**Table 1:** Summary of meteorological variables and TB cases by region, gender, and age group in 22 cities during 2011–2020

Variables	Sum (%)	Mean	SD	Min	P50	Max
**Meteorological variables**						
Humidex		12.66	16.87	-37.72	13.09	46.83
Temperature (℃)		11.41	12.27	-32.32	12.90	34.38
Relative humidity (%)		67.66	14.53	11.74	70.16	96.66
Wind speed (m/s)		2.42	0.93	0.39	2.37	6.82
PM_2.5_ (µg/m^3^)		44.75	30.28	3.13	36.27	246.74
**Overall**	849,676 (100)	73	58	0	56	489
**Region**						
North	327,725 (38.6)	88	56	1	74	405
South	467,867 (55.0)	88	59	0	75	489
Northwest	30,343 (3.57)	29	15	0	26	81
Qinghai-Tibet	23,741 (2.8)	15	10	0	13	50
**Gender**						
Male	579,690 (68.2)	50	40	0	39	340
Female	269,986 (31.8)	23	19	0	17	150
**Age (years)**						
0–15	5,509 (0.7)	0	1	0	0	16
15–40	349,879 (41.2)	30	30	0	21	207
40–65	329,092 (38.7)	28	24	0	22	215
65+	165,196 (19.4)	14	12	0	11	130

**Fig. 2:**
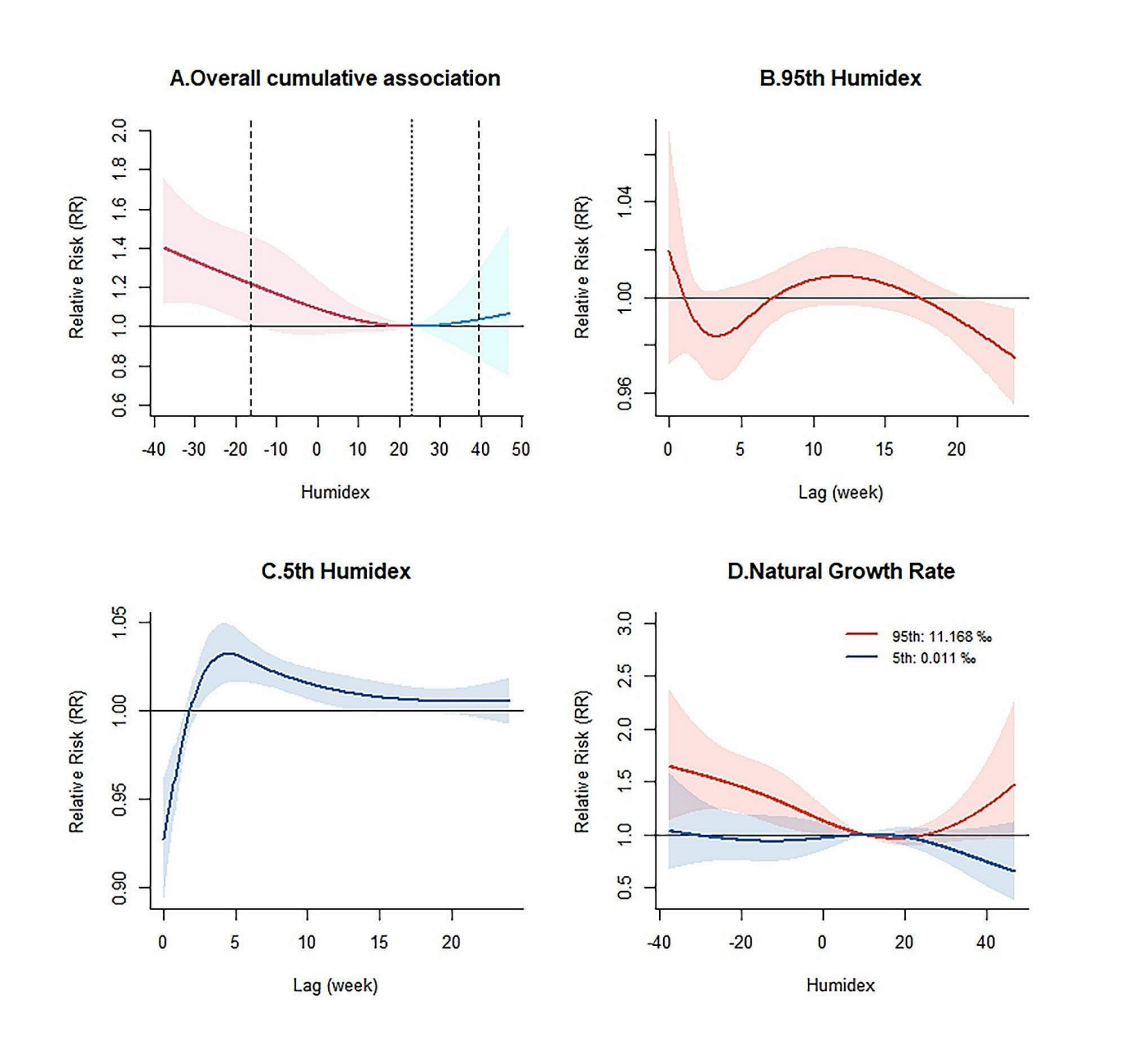
Pooled cumulative relative risks (RR) with 95% Confidence Intervals (CI) of tuberculosis incidence associated with Humidex, over lag 0–24 weeks during 2011–2020 in China. **(A)** Overall cumulative RR at the national level. **(B)** Lagged effects with 95th Humidex. **(C)** Lagged effects with 5th Humidex. **(D)** Cumulative RR in different Natural Growth Rates

Figure 2A shows that humidex and TB incidence have a J-shaped relationship, with the cumulative RR increasing as humidex decreases. An approximate J-shaped exposure-response relationship was also presented in most cities (Figure S1). The curves for temperature and relative humidity are also approximately J-shaped, with decreases in both variables increasing the risk of TB (Figure S2). The maximum RR value is 1.40 (95% CI: 1.11–1.76) at a humidex of -37.72. Fig. 2B, C explore the lagged effects of the 95th and 5th percentile humidex on TB incidence. The lagged impact of the 5th percentile humidex occurs from lag 3 to lag 13, with a peak at lag 5 (RR = 1.03, 95% CI: 1.01–1.05). However, the curve of the effect of the 95th percentile humidex is not statistically significant in 0–20 weeks.

Table 2 shows the multivariate meta-regression models for meta-predictors, as assessed by the multivariate Cochran Q test, *I*^*2*^ statistic, LR test, and Wald test. There was significant heterogeneity, with 49.06% of the variations attributed to the actual difference between the 22 cities (Q = 123.67, *p* < 0.001). This heterogeneity remained statistically significant (*p* < 0.001) even after including different city-specific indicators in the single meta-predictor model. Additionally, the NGR explained part of the heterogeneity, reducing the *I*^*2*^ from 49.06% (intercept-only model) to 39.39%. Both the LR and Wald tests for the NGR were statistically significant.

**Table 2:** Multivariate meta-regression models for meta-predictors

Factors	Cochran Q test			I^2^	LR test		Wald test	
	Q	df	*P*	(%)	Stat	*P*	Stat	*P*
Intercept-only	123.67	63	< 0.001	49.06	-	-	-	-
GDP	109.37	60	< 0.001	45.14	8.66	0.034	3.96	0.265
GDP Per Capita	102.84	60	< 0.001	41.66	8.42	0.038	3.63	0.304
Hospitals	120.18	60	< 0.001	50.08	7.30	0.063	2.00	0.572
Hospital Beds	120.28	60	< 0.001	50.12	7.42	0.060	2.13	0.547
Doctors	120.52	60	< 0.001	50.22	7.58	0.056	2.30	0.513
Population	116.24	60	< 0.001	48.38	10.35	0.016	5.19	0.158
Natural Growth Rate	99.00	60	< 0.001	39.39	11.77	0.008	10.20	0.017
Longitude	120.73	60	< 0.001	50.30	8.19	0.042	2.99	0.393
Latitude	107.86	60	< 0.001	44.37	8.16	0.043	3.48	0.323

Even when different city-specific indicators were included in the single meta-predictor model, the heterogeneity remained statistically significant. A meta-regression model was also used to further explore the exposure-response associations for the 5th and 95th percentiles of the NGR. As shown in Fig. 2D, NGR modification effects were more significant at low humidity than at high humidity. Overall, the impact of a higher NGR was greater than that of a lower one, so that higher NGR could strengthen the association between humidex and TB incidence.

**Fig. 3:**
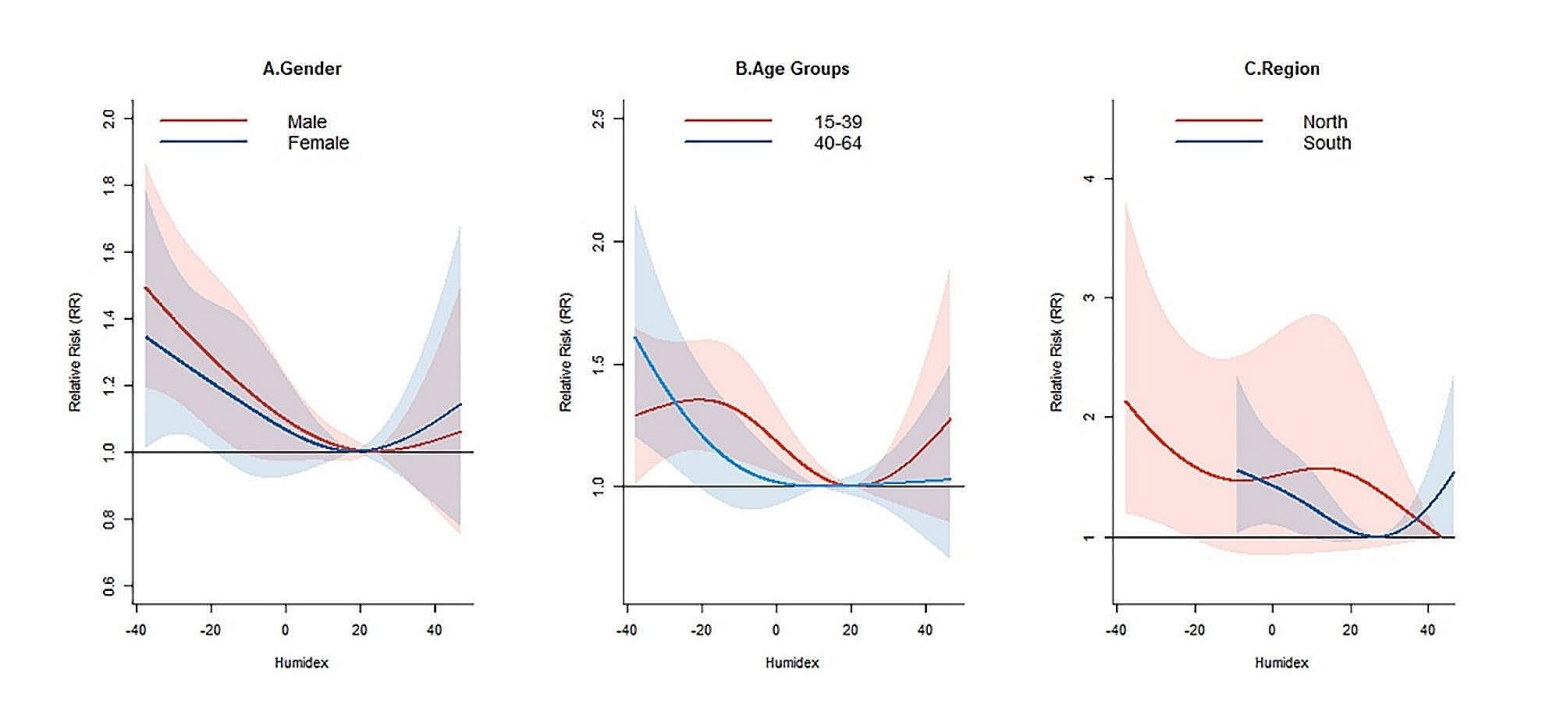
Pooled cumulative relative risks (RR) with 95% Confidence Intervals (CI) of tuberculosis incidence associated with Humidex for different **(A)** gender, **(B)** age groups and **(C)** region, over lag 0–24 weeks during 2011–2020 in China

Figure 3 presents the cumulative associations between humidex and TB incidence in different regions, genders, and age groups. Most of the curves were J-shaped (Fig. 3 A-C), except for the Northwest and Qinghai-Tibet regions (Figure S3), where the curves were not statistically significant. After the multivariate Wald test, the association between humidex and TB incidence was not significantly modified by region, gender, or age group (Table S2).

The multivariate meta-analysis by region, gender, and age group indicated that the associations between humidex and TB incidence were similar across cities in North China, but residual heterogeneity was significant in other regions (Table S3). At the national level, there was significant heterogeneity among cities in the associations between humidex and TB incidence for different gender and age groups, except for the 0–14 age group.

Sensitivity analysis indicated that the model was robust. The results remained reliable when we changed the maximum lag time and the *df* for the time trend (Figure S4A, S4B). Controlling for wind speed and PM_2.5_ did not substantially affect the Humidex-TB association (Figure S4C), and the number of cases in the previous week had no significant effect on the main model (Figure S4D).

## Discussion

To the best of our knowledge, this is the first study to explore the joint effect of temperature and relative humidity on TB incidence in multiple cities in China using the Humidex index and two-stage time series analysis. We also examined heterogeneity by considering city-specific socio-economic, demographic, geographic, and medical service characteristics. The results showed a non-linear relationship between humidex and TB incidence, with a J-shaped association at the national level. Decreasing humidex increased the risk of TB incidence. NGR may affect the Humidex-TB relationship and increase TB incidence.

Low humidex could increase the risk of TB incidence. This finding is consistent with some previous reports in China, although most of these studies explored temperature and relative humidity separately [7, 26, 27]. For instance, studies in Jiangsu [9] and Hong Kong [7] found that low humidity and low temperature increase the risk of TB. There are several reasons for the Humidex-TB association. First, humidex can influence human activity and behavior. When humidex is low (meaning low temperature and low relative humidity), people are more likely to stay indoors and ventilate less, which provides favorable conditions for the survival and transmission of *M. tuberculosis* [28]. Second, humidex can change human health status. Low humidex shortens the sunshine duration, reducing vitamin D synthesis. Studies have found that vitamin D deficiency weakens the immune system, making it more difficult for the body to defend against *M. tuberculosis* infection [28, 29]. Low humidex also dries out the airway mucosa, weakening its ability to defend against pathogens [30, 31]. Third, humidex indirectly regulates urban air quality, which can affect TB incidence. Low humidity prevents suspended particles from condensing and depositing, increasing the airborne transmission of *M. tuberculosis*. High humidex promotes the condensation and deposition of suspended particles, improving air quality [27, 32].

Analysis of heterogeneity at the national level showed that the 22 cities varied significantly in their Humidex-TB relationship. Further analysis suggested that NGR could partly explain this heterogeneity. In this study, a higher NGR was associated with a stronger Humidex-TB relationship. This finding is plausible, given the contagious nature of TB. High urban density and migration can increase the incidence of TB [33]. Additionally, high NGR is often associated with highly urbanized cities, which tend to have higher population density and worse air quality. People living in such environments may be more exposed to pathogens and suffer more health problems [13, 34]. However, there are some conflicting reports about the relationship between socio-economy and TB. Some studies suggest that TB is more likely to occur in poor, low-GDP areas, where health facilities, health surveillance, nutrition, and diet are often inadequate [35, 36]. Given the complex interplay between socioeconomic development, climate, and environmental factors, the relationship between socio-economy and TB deserves further exploration.

Our subgroup analysis found no significant modification effects of region, gender, or age groups on the Humidex-TB relationship. Previous studies have reported that males and older adults may be more vulnerable to TB associated with temperature or relative humidity, due to a complex interplay of biological, social, and economic factors, such as alcohol consumption, smoking, hormones, and nutrition [37, 38]. The discrepancy in results may be due to differences in the study setting, statistical methods, and other factors. Further research is needed to explore the potential modification effects of gender and age on the association between humidex and TB incidence.

This study had several limitations. First, the meteorological data were extracted from the ERA5 reanalysis dataset, which reanalyzes historical meteorological observations with strict quality control. There may have been some introduced measurement bias due to the lack of individual-level exposure data. Second, this ecological study could not establish the causal association between humidex and TB incidence due to the inevitable ecological fallacy and the unavailability of individual-level behavioral data such as indoor conditions. Third, there was an inevitable recall bias as the onset date was estimated by patient recall. However, this study was conducted on a weekly time scale, which can mitigate this limitation to some extent.

In conclusion, this study characterized the associations between humidex and TB incidence in China and found a J-shaped exposure-response relationship. Compared with temperature and relative humidity, humidex may be a better predictor of TB incidence and could enable early prevention and control of TB in China, especially in areas with higher NGRs. The early warning to control TB using humidex should be at least 3 weeks in advance. This study provides important insights for healthcare institutions seeking to develop effective prevention and control strategies for TB incidence.

### Electronic supplementary material

Below is the link to the electronic supplementary material.

Supplementary Material 1

## Data Availability

The datasets used and analyzed during the current study are available from the corresponding author upon reasonable request.

## Abbreviations

TB: Tuberculosis
DLNM: Distributed lag non-linear model
GDP: Gross Regional Product
df: Degrees of freedom
LR: Likelihood ratio
RR: Relative risk
CI: Confidence Intervals
NGR: Natural Growth Rate
