# Supplementary material for: The association between humidex and tuberculosis: a two-stage modelling nationwide study in China
Source: BMC Public Health. 2024 May 11;24:1289. doi: 10.1186/s12889-024-18772-8 (PMC11088084; doi:10.1186/s12889-024-18772-8)
Supplement: Supplementary file 1 — Supplementary Material 1 [file 12889_2024_18772_MOESM1_ESM.docx]

**The association between humidex and tuberculosis: a two-stage modeling nationwide study in China**

Wen Li^1,2†^, Jia Wang^3†^, Wenzhong Huang^4^, Yu Yan^1,2^, Yanming Liu^4^, Qi Zhao^1,2^, Mingting Chen^3*^, Liping Yang^1*^, Yuming Guo^4^, Wei Ma^1,2*^

^1^Department of Epidemiology, School of Public Health, Cheeloo College of Medicine, Shandong University, Jinan, Shandong, China

^2^Shandong University Climate Change and Health Center, Jinan, Shandong, China

^3^National Center for Tuberculosis Control and Prevention, Chinese Center for Disease Control and Prevention, Beijing, China

^4^Climate, Air Quality Research Unit, School of Public Health and Preventive Medicine, Monash University, Melbourne, Australia

**^*^** **Correspondence and requests for reprints should be addressed to**

chenmt@chinacdc.cn (Mingting Chen);

[yliping@sdu.edu.cn](mailto:yliping@sdu.edu.cn) (Liping Yang);

weima@sdu.edu.cn (Wei Ma);

^†^These authors contributed equally to this work.

**Equation S1.** The formula of humidex:

$Humidex=Tem+\frac{5}{9}\{6.112\times{10}^{\left( \frac{7.5\times Tem}{237.7+Tem} \right)}\times\frac{Hum}{100}-10\}$**,**

where *Tem* is temperature; *Hum* is relative humidity


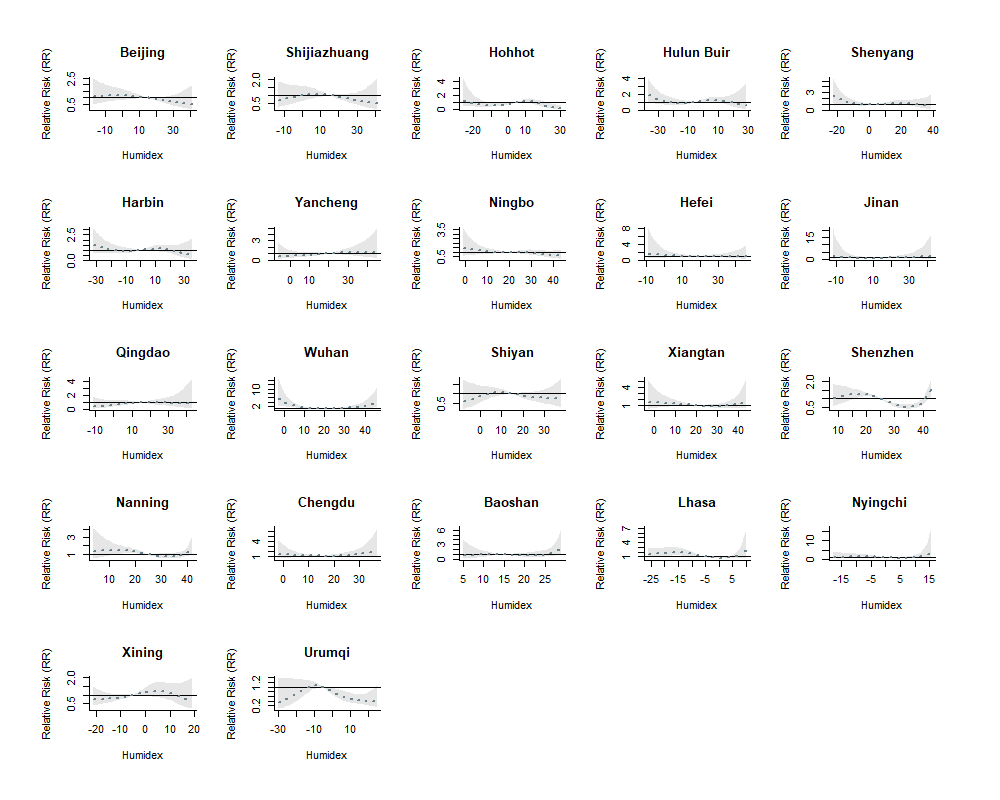


**Figure S1.** Relative risks (RR) with 95% Confidence Intervals (CI) of tuberculosis incidence associated with Humidex, over lag 0-24 weeks during 2011-2020 in each city.


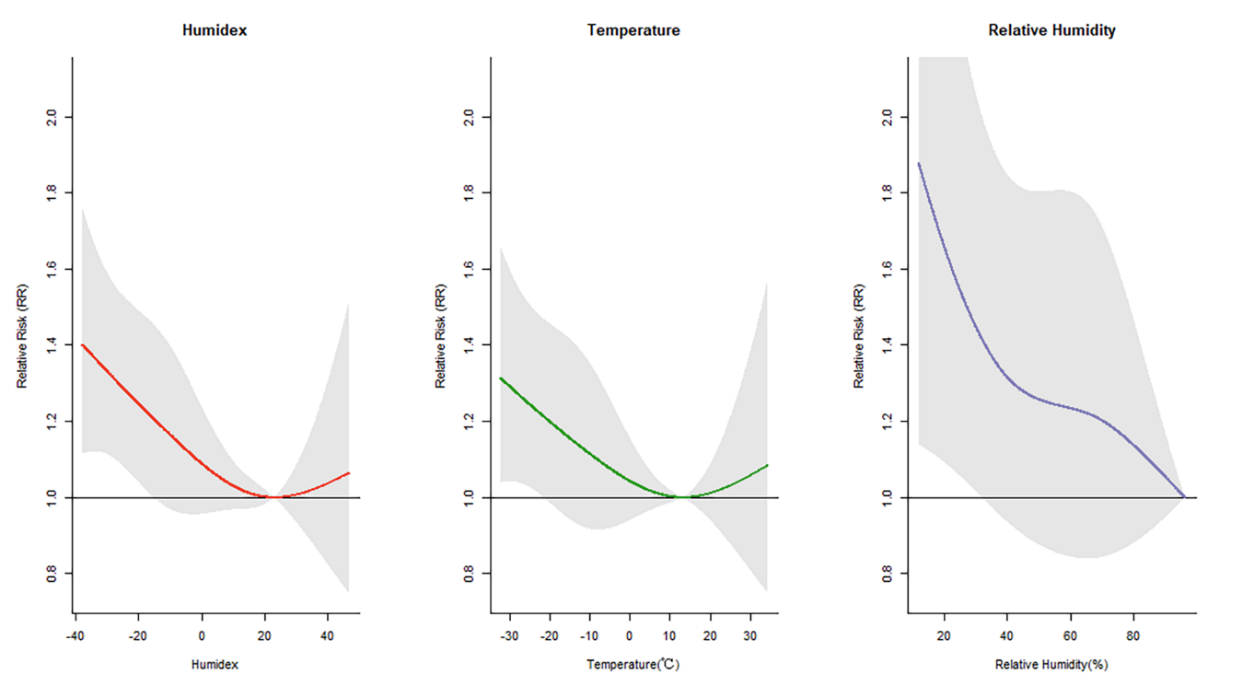


**Figure S2.** Pooled national level overall cumulative relative risks (RR) of humidex, temperature, and relative humidity on TB incidence over lag 0-24 weeks during 2011-2020. Reference: humidex: 17.57; temperature: 11.64℃; relative humidity: 46.56%.


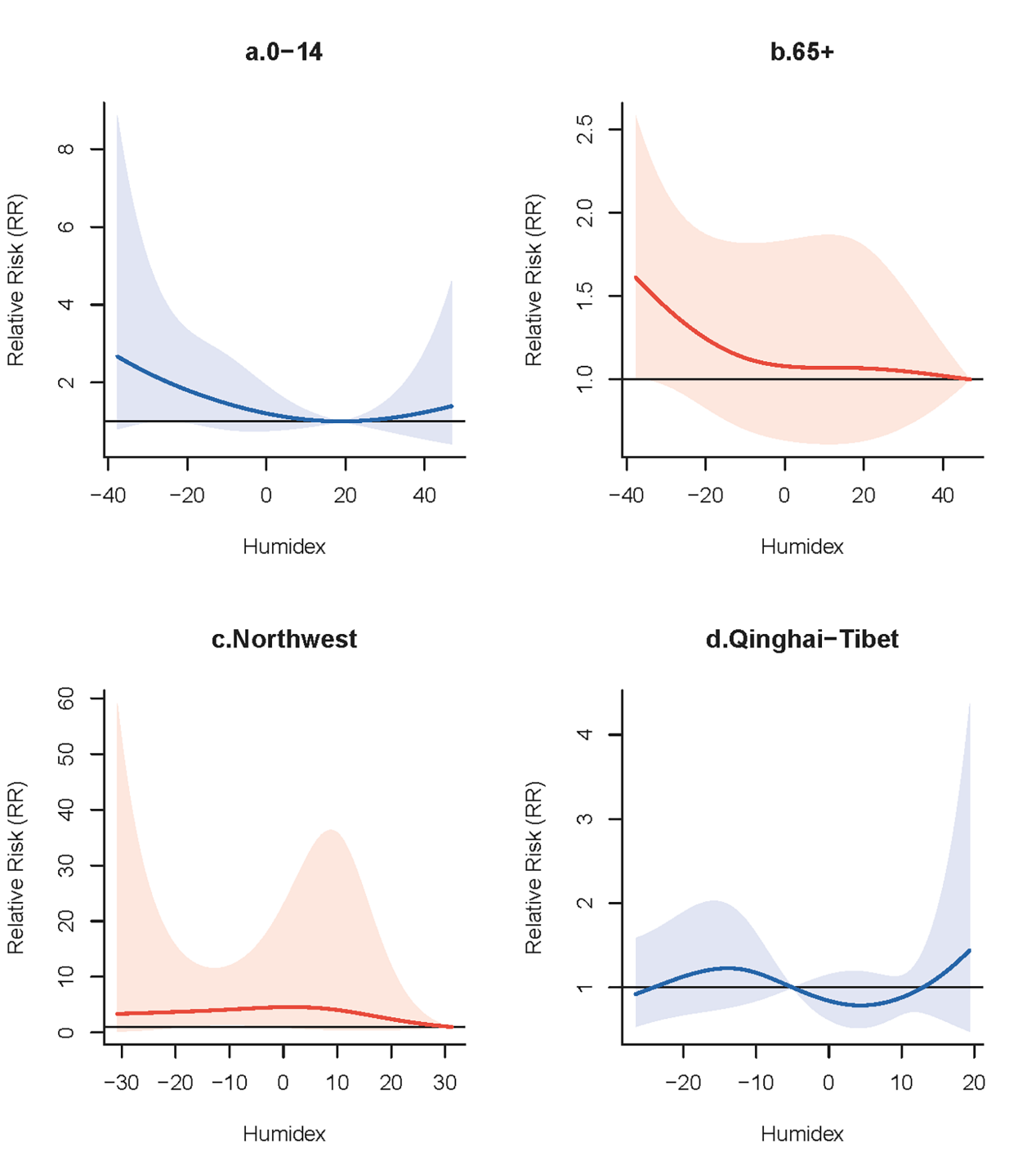


**Figure S3.** Pooled cumulative relative risks (RR) with 95% Confidence Intervals (CI) of tuberculosis incidence associated with Humidex over lag 0-24 weeks during 2011-2020 in China. A. Cumulative RR in the 0-14 age group. B. Cumulative RR in the 65+ age group. C. Cumulative RR in Northwest region. D. Cumulative RR in Qinghai-Tibet region.


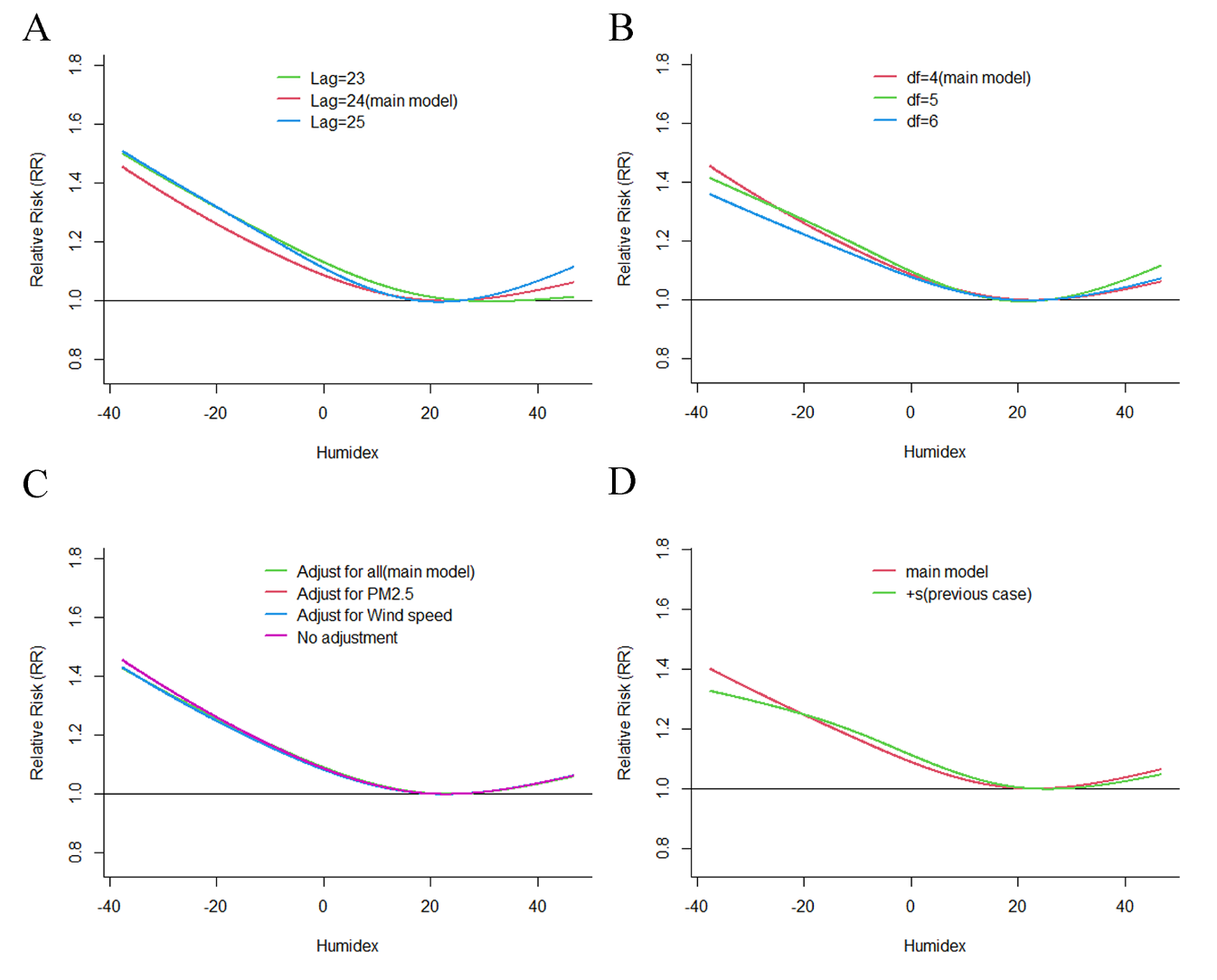


**Figure S4.** Pooled national level overall cumulative relative risks (RR) of humidex on TB incidence when changing the maximum lag time (A), the *df* for the time trend (B), the adjustment factors (C), and adding the *previous case* (D).

**Table S1.** Descriptive statistics of the city-specific characteristics of the 22 cities, 2011-2020.

| City name | MGD | Region | Cases | GDP | GDPP | Hospitals | Hospitals Bed | Doctors | Population | NGR | Longitude | Latitude | PM_2.5_ |
| --- | --- | --- | --- | --- | --- | --- | --- | --- | --- | --- | --- | --- | --- |
| Baoshan | Southwest | South | 8722 | 5838236.4 | 24499.3 | 117.9 | 9859.6 | 4106.1 | 239.8 | 5.1 | 99.1 | 25.1 | 21.54 |
| Beijing | Northern, | North | 77092 | 253455358.7 | 124031.8 | 7262.4 | 683796.6 | 363747.2 | 1333.0 | 5.2 | 116.2 | 39.6 | 59.61 |
| Chengdu | Southwest | South | 64380 | 118213178.5 | 79190.9 | 748.7 | 110479.4 | 54218.3 | 1153.6 | 3.1 | 104.0 | 30.4 | 58.46 |
| Harbin | Northeast | North | 80110 | 52468584.9 | 57671.9 | 399.8 | 67209.6 | 23632.8 | 974.6 | 0.0 | 126.4 | 45.4 | 40.39 |
| Hefei | Jianghuai | South | 43419 | 61679445.7 | 83762.5 | 361.7 | 43512.6 | 19900.0 | 726.2 | 8.0 | 117.2 | 31.5 | 62.62 |
| Hohhot | Inner Mongolia | Northwest | 9805 | 27202056.9 | 93986.0 | 138.7 | 15893.5 | 8498.8 | 237.7 | 5.0 | 111.4 | 40.5 | 40.46 |
| Hulun Buir | Inner Mongolia | North | 16790 | 12576318.6 | 54321.8 | 219.4 | 13066.6 | 7836.3 | 260.5 | 0.4 | 122.0 | 46.0 | 18.53 |
| Jinan | Huanghuai | North | 27559 | 67176711.6 | 91107.7 | 262.1 | 45632.6 | 29194.0 | 641.5 | 6.5 | 117.0 | 36.4 | 79.48 |
| Lhasa | Tibet | Qinghai-Tibet | 7454 | 4061215.0 | 62164.4 | 109.7 | 3159.6 | 2291.4 | 53.0 | 9.4 | 91.1 | 29.4 | 19.36 |
| Nanning | Southern | South | 68868 | 34450520.8 | 50172.0 | 179.0 | 35817.7 | 21643.7 | 696.1 | 8.9 | 108.2 | 22.5 | 33.70 |
| Ningbo | Jiangnan | South | 38950 | 85492848.1 | 115942.0 | 203.4 | 31910.5 | 23479.1 | 587.5 | 2.4 | 121.3 | 29.5 | 33.29 |
| Nyingchi | Tibet | Qinghai-Tibet | 3360 | 1176425.0 | 81003.3 | 28.4 | 765.9 | 582.6 | 21.0 | 11.3 | 94.4 | 29.7 | 17.56 |
| Qingdao | Huanghuai | North | 27297 | 94570420.0 | 108337.3 | 349.8 | 47126.6 | 28587.3 | 785.7 | 3.1 | 120.2 | 36.0 | 58.93 |
| Shenyang | Northeast, | North | 56392 | 64076835.9 | 79571.8 | 307.3 | 58665.9 | 26700.6 | 732.3 | -0.2 | 123.3 | 41.5 | 46.24 |
| Shenzhen | Southern | South | 98604 | 193262141.1 | 186568.0 | 129.5 | 36491.6 | 31099.2 | 364.1 | 19.8 | 114.1 | 22.3 | 35.04 |
| Shijiazhuang | Northern, | North | 42485 | 51209035.6 | 51862.1 | 348.2 | 47121.1 | 31405.5 | 1008.7 | 8.7 | 114.3 | 38.0 | 77.59 |
| Shiyan | Jianghan | South | 27866 | 13572897.7 | 44774.8 | 125.9 | 20797.9 | 9143.6 | 319.7 | 6.2 | 110.5 | 32.4 | 40.80 |
| Urumqi | Northwest | Northwest | 20538 | 26006343.0 | 75681.1 | 154.7 | 27282.7 | 14096.7 | 249.8 | 5.1 | 87.4 | 43.5 | 30.83 |
| Wuhan | Jianghan | South | 65912 | 116801610.0 | 110746.4 | 325.8 | 70201.8 | 33845.6 | 841.3 | 5.9 | 114.2 | 30.4 | 71.59 |
| Xiangtan | Jiangnan | South | 21928 | 16986621.6 | 67402.3 | 92.3 | 14770.8 | 6989.5 | 267.5 | 3.5 | 112.5 | 27.5 | 56.71 |
| Xining | Northwest | Qinghai-Tibet | 12927 | 11041978.0 | 50795.1 | 98.9 | 17352.2 | 9870.5 | 205.8 | 6.2 | 101.5 | 36.4 | 33.79 |
| Yancheng | Jianghuai | South | 29218 | 41170430.0 | 55103.6 | 231.7 | 30172.5 | 16868.3 | 824.7 | 3.4 | 120.2 | 33.4 | 47.98 |

Note: MGD: Meteorological Geographical Divisions; GDP: Gross Regional Product (CNY); GDPP: GDP Per Capita (CNY); Hospitals: Number of Hospitals; Hospital Beds: Number of Hospitals Beds; Doctors: Number of Licensed (Assistant) Doctors; Population: Annual Average Population (10 000 persons); NGR: Natural Growth Rate (‰); Longitude (degree east); Latitude (degree north).

**Table S2.** The cumulative relative risk of TB incidence associated with low (5^th^ percentile) and high (95^th^ percentile) humidex, compared with 75^th^ percentile in different groups.

| Factors | 5th, 95th of Humidex | High humidex effect | Low humidex effect | *P* value for difference |
| --- | --- | --- | --- | --- |
| **Region** |  |  |  |  |
| North | -22.3, 34.9 | 1.2 (0.95, 1.53) | 1.63 (1.01, 2.64) | ref |
| South | 0.8, 41.4 | 1.3 (0.99, 1.71) | 1.42 (1.1, 1.83) | 0.106 |
| Northwest | -20.4, 23.5 | 1.87 (0.58, 6.01) | 3.68 (0.84, 16.18) | 0.192 |
| Qinghai-Tibet | -17.3, 13.6 | 1.31 (0.66, 2.62) | 1.52 (0.61, 3.77) | 0.181 |
| Total | -16.2, 39.5 | 1.04 (0.83, 1.29) | 1.22 (1.01, 1.46) |  |
| **Gender** |  |  |  |  |
| Male | -16.2, 39.5 | 1.03 (0.83, 1.28) | 1.24 (1.03, 1.5) | 0.787 |
| Female | -16.2, 39.5 | 1.09 (0.85, 1.4) | 1.18 (0.97, 1.43) | ref |
| **Age(years)** |  |  |  |  |
| 0-14 | -16.2, 39.5 | 1.23 (0.55, 2.71) | 1.66 (0.89, 3.09) | 0.631 |
| 15-40 | -16.2, 39.5 | 1.16 (0.89, 1.5) | 1.34 (1.13, 1.59) | 0.430 |
| 41-65 | -16.2, 39.5 | 1.02 (0.79, 1.31) | 1.15 (0.95, 1.4) | 0.993 |
| 65+ | -16.2, 39.5 | 1.02 (0.85, 1.23) | 1.19 (0.78, 1.84) | ref |

**Table S3.** The multivariate meta-analysis by region, gender, and age group.

| Groups | Q | *df* | *p* | *I^2^* (%) | AIC | BIC |
| --- | --- | --- | --- | --- | --- | --- |
| **Overall** | 123.67 | 63 | <0.001 | 49.06 | 176.14 | 195.43 |
| **Region** |  |  |  |  |  |  |
| North | 18.44 | 18 | 0.427 | 2.38 | 58.20 | 66.21 |
| South | 48.63 | 27 | 0.007 | 44.48 | 83.00 | 94.66 |
| Northwest | 15.01 | 3 | 0.002 | 80.01 | 27.12 | 19.01 |
| Qinghai-Tibet | 14.14 | 6 | 0.028 | 57.56 | 32.89 | 31.02 |
| **Gender** |  |  |  |  |  |  |
| Male | 113.77 | 63 | <0.001 | 44.63 | 179.82 | 199.11 |
| Female | 105.01 | 63 | 0.001 | 40.01 | 196.04 | 215.33 |
| **Age(years)** |  |  |  |  |  |  |
| 0-15 | 62.72 | 63 | 0.486 | -0.44 | 361.00 | 380.29 |
| 15-40 | 97.62 | 63 | 0.003 | 35.47 | 189.04 | 208.32 |
| 40-65 | 98.60 | 63 | 0.003 | 36.11 | 186.87 | 206.15 |
| 65+ | 139.46 | 63 | <0.001 | 54.82 | 234.85 | 254.14 |

AIC: Akaike Information Criterion;

BIC: Bayesian Information Criterion;
